# Alternative lengthening of telomeres is the major telomere maintenance mechanism in astrocytoma with isocitrate dehydrogenase 1 mutation

**DOI:** 10.1007/s11060-020-03394-y

**Published:** 2020-01-20

**Authors:** Monica Sofia Ventura Ferreira, Mia Dahl Sørensen, Stefan Pusch, Dagmar Beier, Anne-Sophie Bouillon, Bjarne Winther Kristensen, Tim Henrik Brümmendorf, Christoph Patrick Beier, Fabian Beier

**Affiliations:** 1 Department of Haematology, Oncology, Medical Faculty, RWTH Aachen, Pauwelsstrasse 30, 52074 Aachen, Germany; 2 Department of Pathology, University Hospital Odense, Sdr. Boulevard 29, 5000 Odense, Denmark; 3 Department of Clinical Research, University of Southern Denmark, Sdr. Boulevard 29, 5000 Odense, Denmark; 4 Department of Neuropathology, University of Heidelberg, Heidelberg, Germany; 5 Clinical Cooperation Unit Neuropathology, German Cancer Research Center (DKFZ), Heidelberg, Germany; 6 German Cancer Consortium (DKTK), Heidelberg, Germany; 7 Department of Neurology, University Hospital Odense, Sdr. Boulevard 29, 5000 Odense, Denmark

**Keywords:** Isocitrate dehydrogenase, D2HG, Telomerase, Telomere length, Q-FISH, TERT promoter, ALT, ATRX

## Abstract

**Purpose:**

Isocitrate dehydrogenase 1 (IDH1) mutations are associated with improved survival in gliomas. Depending on the IDH1 status, TERT promoter mutations affect prognosis. IDH1 mutations are associated with alpha-thalassemia/mental retardation syndrome X-linked (ATRX) mutations and alternative lengthening of telomeres (ALT), suggesting an interaction between IDH1 and telomeres. However, little is known how IDH1 mutations affect telomere maintenance.

**Methods:**

We analyzed cell-specific telomere length (CS-TL) on a single cell level in 46 astrocytoma samples (WHO II-IV) by modified immune-quantitative fluorescence in situ hybridization, using endothelial cells as internal reference. In the same samples, we determined IDH1/TERT promoter mutation status and ATRX expression. The interaction of IDH1^R132H^ mutation and CS-TL was studied in vitro using an IDH1^R132H^ doxycycline-inducible glioma cell line system.

**Results:**

Virtually all ALT^positive^ astrocytomas had normal TERT promoter and lacked ATRX expression. Further, all ALT^positive^ samples had IDH1^R132H^ mutations, resulting in a significantly longer CS-TL of IDH1^R132H^ gliomas, when compared to their wildtype counterparts. Conversely, TERT promotor mutations were associated with IDH^wildtype^, ATRX expression, lack of ALT and short CS-TL. ALT, TERT promoter mutations, and CS-TL remained without prognostic significance, when correcting for IDH1 status. In vitro, overexpression of IDH^R132H^ in the glioma cell line LN319 resulted in downregulation of ATRX and rapid TERT-independent telomere lengthening consistent with ALT.

**Conclusion:**

ALT is the major telomere maintenance mechanism in IDH^R132H^ mutated astrocytomas, while TERT promoter mutations were associated with IDH^wildtype^ glioma. IDH1^R132H^ downregulates ATRX expression in vitro resulting in ALT, which may contribute to the strong association of IDH1^R132H^ mutations, ATRX loss, and ALT.

**Electronic supplementary material:**

The online version of this article (10.1007/s11060-020-03394-y) contains supplementary material, which is available to authorized users.

## Introduction

Gliomas are the most common primary brain tumors in adults and represent a histologically defined entity with a high molecular heterogeneity determining prognosis and response to therapy [1, 2]. Along this line, determination of isocitrate dehydrogenase (IDH) 1 mutation status became mandatory in the updated 2016 WHO classification [3]. IDH1 mutations are driver mutations of low-grade gliomas and found in 80% [4] but are not detectable in primary glioblastoma (GBM) [2, 5]. The most common IDH1 mutations in glioma (> 95%) result in an amino acid substitution at arginine 132 (R132), which resides in the enzyme’s active site [4]. Despite the abundance of evidence in support of a major pathophysiological and prognostic role of IDH1 mutations [6–8], the precise mechanism of how IDH1 mutations modulate malignancy is still not completely understood. While IDH1 has a major role in the citric acid cycle, R132H mutation (IDH^R132H^) results in a gain of function; IDH1^R132H^ catalyzes conversion of alpha-ketoglutarate into the oncometabolite D-2-hydroxyglutarate (D2HG) [9]. D2HG inhibits dioxygenases that depend on alpha-ketoglutarate, like Ten-eleven translocation methylcytosine dioxygenase 1 (TET1) and histone-lysine demethylases [10]; this results in increased CpG island methylation [11] and a stable reshaping of the epigenome (CpG island methylation phenotype, CIMP) changing transcriptional programs and altering the differentiation state [12].

Telomeres determine the proliferative capacity of mammalian cells. Telomere length (TL) shortens with each cell division until cell proliferation is arrested once the maximal number of cell divisions is reached (“Hayflick limit”). In case of further replication, cells undergo chromosomal instability and induction of apoptosis. Expression of human telomerase reverse transcriptase (TERT) allows for the stabilization and elongation of telomeres. Telomere maintenance mechanisms (TMM) are necessary for immortality of cancer cells by overcoming genetic instability associated with critical telomere shortening [13].

TERT promoter mutations (TERTp^mut^) are common in glioma and found in 80% of all primary GBM [14–16], representing one possible TMM. TERTp^mut^ disrupt the tight transcriptional suppression of TERT in somatic cells resulting in increased TERT expression and telomerase activity in vitro in glioma [17].

A mechanism of homologous recombination [18] to maintain TL, known as “alternative lengthening of telomeres” (ALT), was also identified in gliomas. About 20–63% of adult low-grade glioma and 11% of adult GBM use ALT as an additional mechanism for telomere maintenance [19, 20]. Dysfunction of the α-thalassemia/mental retardation syndrome X-linked (ATRX)/death-associated protein 6 (DAXX) complex is known to result in ALT along with more widespread genomic destabilization [21–23]. ATRX and DAXX are central components of a chromatin-remodeling complex required for the incorporation of H3.3 histone proteins into the telomeric regions of chromosomes [24, 25]. 75% of grade II–III astrocytomas and secondary GBM harbor ATRX abberations [26–28] linking IDH1 mutations with ATRX and ALT.

Despite the strong associations observed, few data is available how the different pathways interact on telomere maintenance. The aim of this study was to investigate the interplay among IDH1, TL, ATRX and TERT/ALT using a newly developed distinctive methodology that allowed determination of glioma cell-specific telomere length (CS-TL) on a single cell level.

## Materials and methods

### Patients

Tissue samples from 46 astrocytoma patients were included for the study. The Regional Committee on Health Research Ethics approved the study for Southern Denmark (S2DO90080) and Danish Data Protection Agency (file number: 2009-41-3070) and all patients provide informed consent. The use of tissue was not prohibited by any patient according to the Danish Tissue Application Register. All methods were performed in accordance with the relevant guidelines and regulations. All patients underwent primary surgery between 1991 and 2005 at the Department of Neurosurgery, Odense University Hospital, Denmark. All cases were independently reviewed and reclassified by M.D.S and B.W.K. (senior neuropathologist) according to the 2016 WHO guidelines [2] as described in [29]. Clinical data were extracted from the respective electronic patient journal. Clinical and neuropathological characteristics of the astrocytoma patients are shown in Table 1.

**Table 1:** Patient characteristics and pathology

	Diffuse astrocytoma	Anaplastic astrocytoma	Glioblastoma	All
Patients (n)	24	9	13	46
Age				
Mean	45.2	47.6	65.3	51.4
Range	2.6–78.5	29.5–71.0	49.4–77.5	2.6–78.5
Sex (n)				
Male	14 (58.3%)	3 (33.3%)	10 (76.9%)	27 (58.7%)
Female	10 (42.7%)	6 (66.7%)	3 (23.1%)	19 (41.3%)
Status (n)				
Alive	3 (12.5%)	0 (0%)	0 (0%)	3 (6.5%)
Dead	21 (87.5%)	9 (100%)	13 (100%)	43 (93.5%)
Overall survival (months)				
Median	76.9	24.0	7.5	32.6
Range	3.4–280.0	4.7–48.8	1.5–34.2	1.5–280.0
IDH1 status (n)				
Mutated	18 (75.0%)	4 (44.4%)	1 (7.7%)	23 (50.0%)
Wildtype	6 (25.0%)	5 (55.6%)	12 (92.3%)	23 (50.0%)
ATRX status (n)				
Loss	20 (83.3%)	4 (44.4%)	4 (30.8%)	28 (60.9%)
Retained	4 (16.7%)	5 (55.6%)	9 (69.2%)	18 (39.1)
1P/19Q status (n)				
CO-DEL	0	0	0	0
1P-DEL	0	1 (11.1%)	1 (7.7%)	2 (4.3%)
19-DEL	0	0	0	0
NON-DEL	5 (20.8%)	2 (22.2%)	2 (15.3%)	9 (19.6%)
Not determined	19 (79.2%)	6 (66.7%)	10 (76.9%)	35 (76.0%)
hTERT promoter mutation				
Mutated	6 (25.0%)	3 (33.3%)	9 (69.2%)	18 (39.1%)
C228T	5 (83.3%)	2 (66.7%)	8 (88.9%)	15 (83.3%)
C250T	1 (16.7%)	1 (33.3%)	1 (11.1%)	3 (16.7%)
None	17 (75.0%)	5 (55.6%)	4 (30.8%)	26 (56.5%)
Not determined	1 (4.1%)	1 (11.1%)	0	2 (4.3%)
ALT status				
Negative	12 (50%)	5 (55.6%)	12 (92.3%)	29 (63.0%)
Positive	12 (50%)	4 (44.4%)	1 (7.7%)	17 (37.0%)
KI67 proliferation rate				
Mean (%)	3.3	9.7	10.2	6.7
Range (%)	0–30	3–20	2–35	0–35
CS-TL				
Median	11.8	13.6	3.5	11.0
Range	− 1.1 to 36.7	− 3.9 to 31.5	− 6.0 to 25.2	− 6.0 to 36.7

### Immunohistochemistry

Formaldehyde-fixed paraffin embedded (FFPE) sections of three µm from pre-surgery tissue biopsies were used for this study. FFPE sections were stained as described previously using primary antibodies against IDH1^R132H^ (H09, 1:100, Dianova, Germany) [30] and ATRX [29] (HPA001906, 1:100, Atlas Antibodies, Sweden) epitopes.

### DNA extraction, polymerase chain reaction and mutational analysis by sequencing

Mutations in the TERT promoter region were identified by PCR and Sanger sequencing as described previously [31]. The detailed protocol can be found in the Supplementary Materials and methods.

### Cell culture, proliferation and clonogenicity

For the cell culture experiments the doxycycline-inducible GBM cell line LN319 expressing IDH1 wildtype (IDH1^WT^) and IDH1^R132H^ was used. Cells were cultured in Dulbecco’s Modified Eagle Medium (DMEM) (Gibco, Germany) supplemented with 10% tetracycline-free fetal calf serum (FCS) (Clontech, USA) and standard antibiotics (Gibco, Germany). 1 µM doxycycline was used (Sigma Aldrich, Germany) to induce expression. Cell proliferation was assessed using the CellTiter-Blue Assay (Promega, Germany) as described previously [32] using the FLUOstarOPTIMA (BMG Labtech, Germany) fluorometer.

For colony-forming unit assays, 2500 cells/well were seeded in a 6-well format for 10 days before colonies were fixed and stained with Cristal Violet (Sigma, Germany). For agar assays, 8000 cells/well were seeded in a 6-well format and incubated for 3 weeks before cells were stained with Cristal Violet. Images were acquired with a Cool Snap™ HQ2 digital camera (Photometrics, USA) on an Axiophot 2 microscope (Carl Zeiss, Germany). Quantification was done using ImageJ software (open source). Results are means of three repeated experiments.

### (D)-2-hydroxyglutarate (DGH2) assay

The DGH2 assay used was based on an enzymatic assay as previously described [9]. The detailed protocol can be found in the Supplementary Materials and methods.

### RNA extraction, cDNA synthesis and mRNA expression

Determination of TERT and ATRX mRNA expression was carried out as described presviously[32]. Gene expression is expressed in fold change according to the $2^{{ - \Delta \Delta {\text{c}}_{\text{t}} }}$ method. Additional information can be found in the Supplementary Materials and methods.

### Quantitative fluorescence in situ hybridization (Q-FISH)

TL analysis was done by a modified protocol of immuno-quantitative fluorescence in situ hybridization (Q-FISH) as previously described [31–35]. FFPE sections of the cohort were deparaffinized and rehydrated before antigen retrieval in 10 mM citrate buffer (pH 6.0). Slides were permeabilized with 0.2% Triton X-100 and blocked for 30 min in serum-free buffer (Rotiblock 1:10, Roth, Germany). Actin fibers were first stained with primary antibody mouse anti-human alfa-SMA (1:200, DAKO, Germany) and a goat anti-mouse Alexa Fuor 633 (1:100, Thermo Fisher, Germany) as secondary antibody. Next, cells were post-fixed in formalin for 30 s and dehydrated with increasing ethanol series before telomere staining. For cells in culture, cells were recovered from culture, fixed in methanol:acetic acid (3:1), cytospin, air dried and dehydrated with ethanol before telomeres were stained. Telomere staining consisted in providing a hybridization mixture containing the Cy3-(C3TA2) peptide nucleic acid (PNA) probe (Panagene, South Korea) to the slides for 3 min at 85 °C for DNA denaturation. Slides were then hybridized for 2 h at room temperature in a humidified chamber. Next, slides were washed with a formamide-based buffer, DAPI stained, and mounted with Vectashield antifade mounting medium (Vector Labs, USA). Fluorescence was acquired with the high-resolution laser-scanning microscope LSM710 (Zeiss, Germany). H&E stained sections were analyzed in parallel for all cases to identify tumor areas. Fluorescent image capture was done with ×63 optical magnification and ×1.2 zoom. A multi-tracking mode of 0.5 μm-steps was used to acquire images of DAPI, Cy3 and Alexa Fluor 633 stainings. Maximum projection of five single consecutive steps of 1.2 µm each was done for TL quantification using Definiens software (Definiens, Germany). Nuclei and telomeres were detected based on the respective DAPI and Cy3 intensity. Alfa-SMA was used to identify endothelial cells that were used as an internal control to correct for TL inter-individual variability [32–38]. A mean number of 150 tumor cells and 100 endothelial cells were assessed per case. To determine the tumor cell-specific telomere length (CS-TL), the difference (Δ telomere length) between the TL of astrocytoma cells and the TL of endothelial cells was calculated and designated in arbitrary units (a.u.) of fluorescence.

### ALT assessment

All astrocytoma cases were assessed for the presence of ALT phenotype using telomere Q-FISH staining. ALT positivity was identified by large, ultrabright, clumpy, intranuclear foci of telomere FISH signals, as previously described [20, 21]. A tumor was defined as ALT-positive, when the following two criteria were fullfilled: (1) the presence of ultra-bright intranuclear foci of telomere FISH signals (ALT-associated telomeric foci), with integrated total signal intensities for individual foci being > 10-fold the mean signal intensities per cell of all telomeric signals from endothelial cells within the same case and (2) the number of cells with ALT-associated telomeric foci being 1% or more of the total number of tumor cells assessed per case [20, 21].

### ATRX and TERT Immunofluorescence and western blotting

Immunofluorescence for ATRX and TERT followed previously published protocols [32, 35, 37]. Western blot was carried out according to previous published standard protocols. The detailed protocol can be found in the Supplementary Materials and methods.

### Statistical analysis

The data was collected via Microsoft Excel 2007 and analyzed using Stata version 15 (StataCorp LP, USA) or GraphPad Prism 5.0 (GraphPad Software Inc, USA). ANOVA with Bonferroni correction was used for comparison of more than two groups. Student’s unpaired *t* test was used to compare differences between two groups. Fisher’s exact test was used to analyze categorical data. Overall survival was defined from the day of initial surgery until death or date of censoring (July 1st, 2018). Survival data were analysed and we using Kaplan–Meier and the multivariate Cox regression model to adjust for age, WHO grade, and IDH1 status. Survival curves were compared using the log-rank test.

## Results

### Association of IDH1^R132H^ mutations and telomere length

**Fig. 1:**
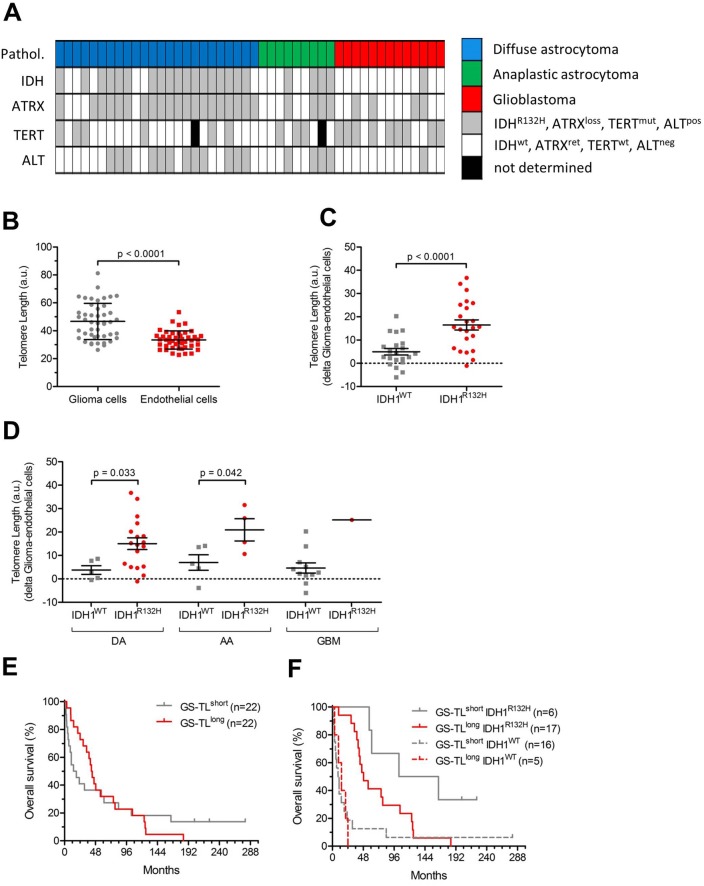
Glioma cell-specific telomere length in IDH1^WT^ and IDH1^R132H^ glioma. **a** Overview of biomarkers status in glioma patients (n = 46). **b** Telomere length in glioma vs endothelial cells. **c** Telomere length (in arbitrary units, a.u.) in IDH1^WT^ and IDH1^R132H^ glioma samples; **d** Telomere length (in a.u.) in IDH1^WT^ and IDH1^R132H^ glioma stratified by WHO tumor grade (*DA* diffuse astrocytoma, *AA* anaplastic astrocytoma, *GBM* gliobastoma multiform); **e** Kaplan–Meier survival curves (in months) of patients with CS-TL below (CS-TL^short^) or above median; **f** Kaplan–Meier survival curves (in months) of patients with CS-TL below (CS-TL^short^) or above median stratified based on IDH1 mutational status

The incidence of IDH1^R132H^ mutations in our cohort was 50% (Fig. 1a, Table 1). Single cell-based telomere analysis using modified immuno-Q-FISH (Supplementary Figure S1A, B) revealed significantly longer TL in astrocytoma as compared to endothelial cells (p < 0.0001) and a substantial inter-individual variability (Fig. 1b). To overcome this issue, we used the endothelial cells as internal control, which allowed correction of the TL of astrocytoma cells for age and for inter-individual variability (i.e. CS-TL). The CS-TL was significantly longer in IDH1^R132H^-mutated tumors as compared to IDH1^WT^ tumors (Fig. 1c) irrespective of tumor grade (Fig. 1d), while there were no significant differences among the different tumor types (p = 0.19, Supplementary Figure S1C). IDH1^R132H^ mutation was significantly associated with improved patient survival (HR 0.28; 95% CI 0.14–0.50; p < 0.001, Supplementary Figure S1C) with similar tendency (HR 0.47; 95% CI 0.21–1.08; p = 0.076) when adjusting for age and WHO grade. CS-TL did not significantly correlate with prognosis (HR 0.98; p = 0.95) (Fig. 1e). However, when dichotomizing patients based on IDH1 status and CS-TL, patients with IDH1^R132^-mutated tumors and long CS-TL had significantly poorer survival than patients with IDH1^R132^-mutated tumors and short CS-TL (HR 1.80; 95% CI 1.03–3.16; p = 0.030, Fig. 1F). No significant prognostic value was found for patients with IDH^WT^ tumors (HR 0.98; 95% CI 0.59–1.65; p = 0.95, Fig. 1f).

### hTERT promoter mutations, IDH1 mutations, and telomere length

**Fig. 2:**
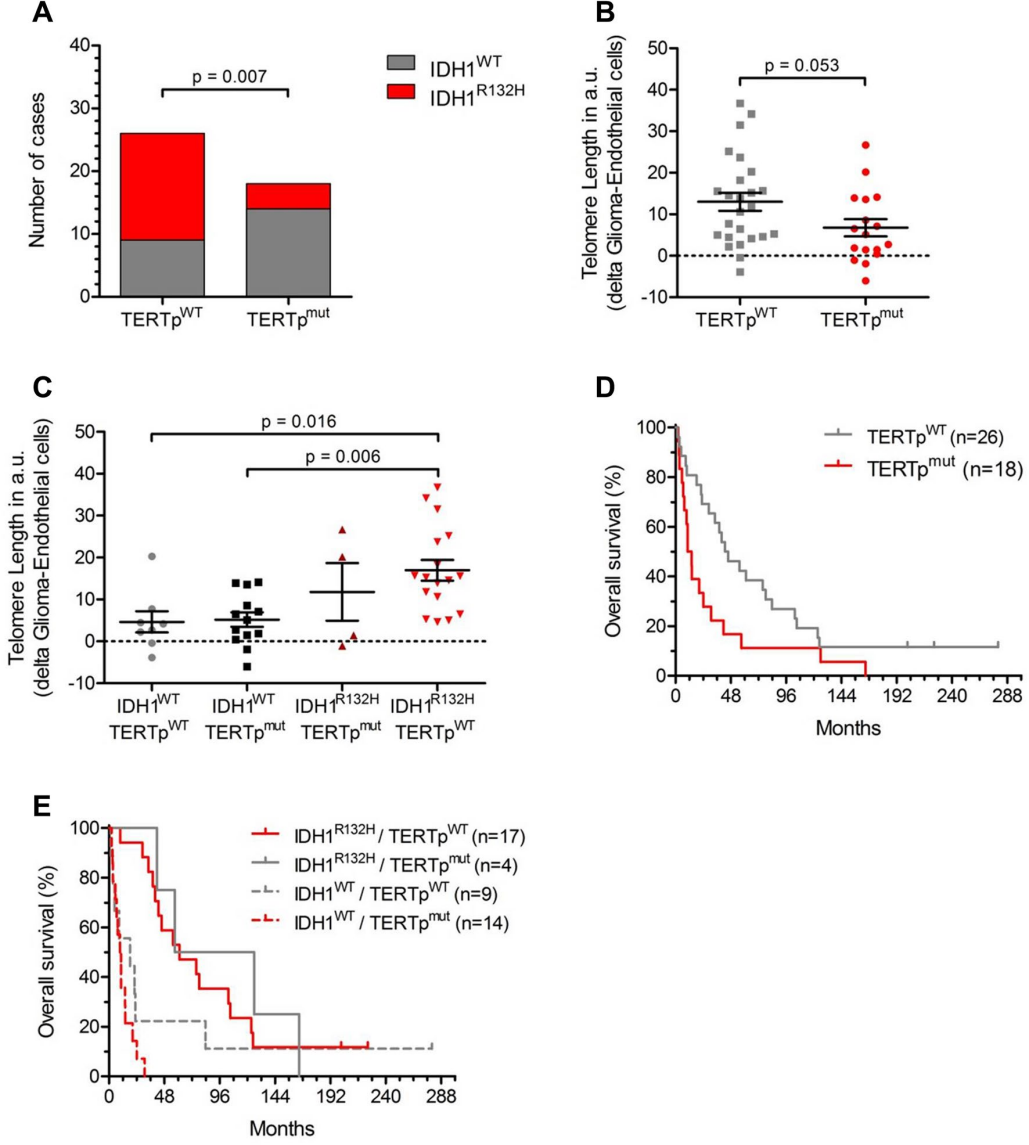
TERT promotor, glioma cell-specific telomere length and IDH status. **a** Distribution of IDH1^WT^ and IDH1^R132H^ among the TERTp^mut^ and TERTp^WT^ patients (p = 0.001, X^2^-test). **b** Glioma-specific telomere length (CS-TL, in arbitrary units, a.u.) in TERTp^WT^ and TERTp^mut^ tumors. **c** CS-TL (in a.u.) of glioma patients according to IDH1 and TERTp^mut^ status. **d** Kaplan–Meier survival curves of TERTp^WT^ and TERTp^mut^ patients. **e** Kaplan–Meier survival curves of TERTp^WT^ and TERTp^mut^ patients stratified after IDH1 status

The incidence of TERTp^mut^ was 43% of which 84% harbored a C228T point mutation and 16% had a C250T point mutation (Supplementary Figure S2A) (Table 1). The presence of TERTp^mut^ was associated with IDH1^WT^ (p = 0.007) (Fig. 2a).

TERTp^mut^ tumors had slightly shorter CS-TL than TERTp^WT^ tumors (p = 0.05) (Fig. 2b). When analyzing CS-TL according to IDH1 and TERTp^mut^ status, we confirmed the increased CS-TL of IDH1^R132^ tumors but did not found significant differences of CS-TL beween IDH1^WT^/TERTp^WT^ and IDH1^WT^/TERTp^mut^ or IDH1^R132H^/TERTp^WT^ and IDH1^R132H^/TERTp^mut^ tumors (Fig. 2c).

TERTp status was significantly associated with reduced survival (HR 2.05; 95% CI 1.10–3.86; p = 0.022) in the entire cohort (Fig. 2d). However, in patients with an IDH1^R132H^ tumor, TERTp status did not significantly associate with survival (Fig. 2e), and multivariate analysis unveiled that TERTp status was not an independent prognostic factor when adjusting for age, WHO grade, and IDH1 status (HR 1.06; 95% CI 0.50–2.24; p = 0.87).

### ALT, ATRX mutations and telomere length

**Fig. 3:**
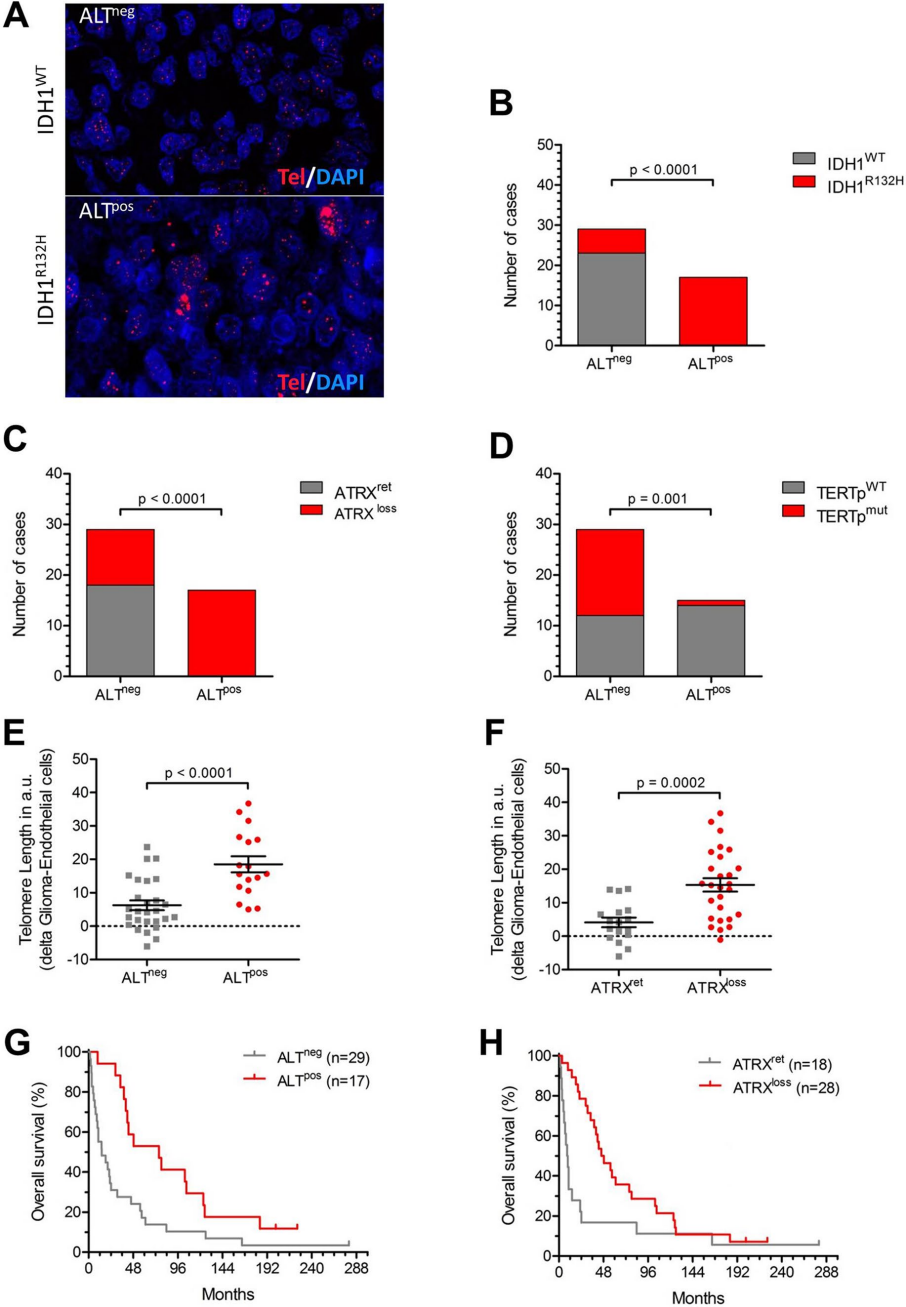
ATRX, alternative lengthening and survival. **a** Representative images showing Q-FISH stained gliomas with (ALT^pos^) and without ALT (ALT^neg^, magnification: 756x). **b** Number of IDH1^WT^ and IDH1^R132^ patients stratified according to ALT status. **c** Number of ATRX^WT^ and ATRX^mut^ patients stratified according to ALT status. **d** Number of TERTp^WT^ and TERTp^mut^ patients stratified according to ALT status. **e** Glioma-specific telomere length (CS-TL in arbitrary units, a.u.) in ALT^neg^ and ALT^pos^ tumors. **f** Glioma-specific telomere length (CS-TL in arbitrary units, a.u.) in tumors with retained (ATRX^ret^) ATRX and in tumors with ATRX loss (ALT^loss^). **g** Kaplan–Meier survival curves (in months) of glioma patients according to ALT status. **h** Kaplan–Meier survival curves (in months) of glioma patients according to ATRX mutational status

Using telomere FISH, we studied the presence of ALT (ALT^pos^) on a single cell level in our cohort (Fig. 3a). 37% of the patients had ALT^pos^ tumors (Table 1) of which 100% also had an IDH1^R132H^ mutation (Fig. 3b). Further, loss of ATRX expression (Fig. 3c) and TERTp^WT^ (Fig. 3d) were associated with the presence of ALT. Both, presence of ALT and loss of ATRX expression were significantly associated with longer telomeres (p < 0.001, Fig. 3e, f) and improved survival (HR 0.42; 95% CI 0.22–0.81; p = 0.007 and HR 0.46; 95% CI 0.4–0.85; p = 0.012, respectively; Fig. 3g, h). However, this association disappeared when adjusting for age, WHO grade and IDH1 status i a multi-variate analysis (ALT status: HR 0.62; 95% CI 0.23–1.72; p = 0.36; ATRX status: HR 1.12; 95% CI 0.43–2.93; p = 0.82).

### In vitro overexpression of mutant IDH^R132H^ in a GBM cell line

**Fig. 4:**
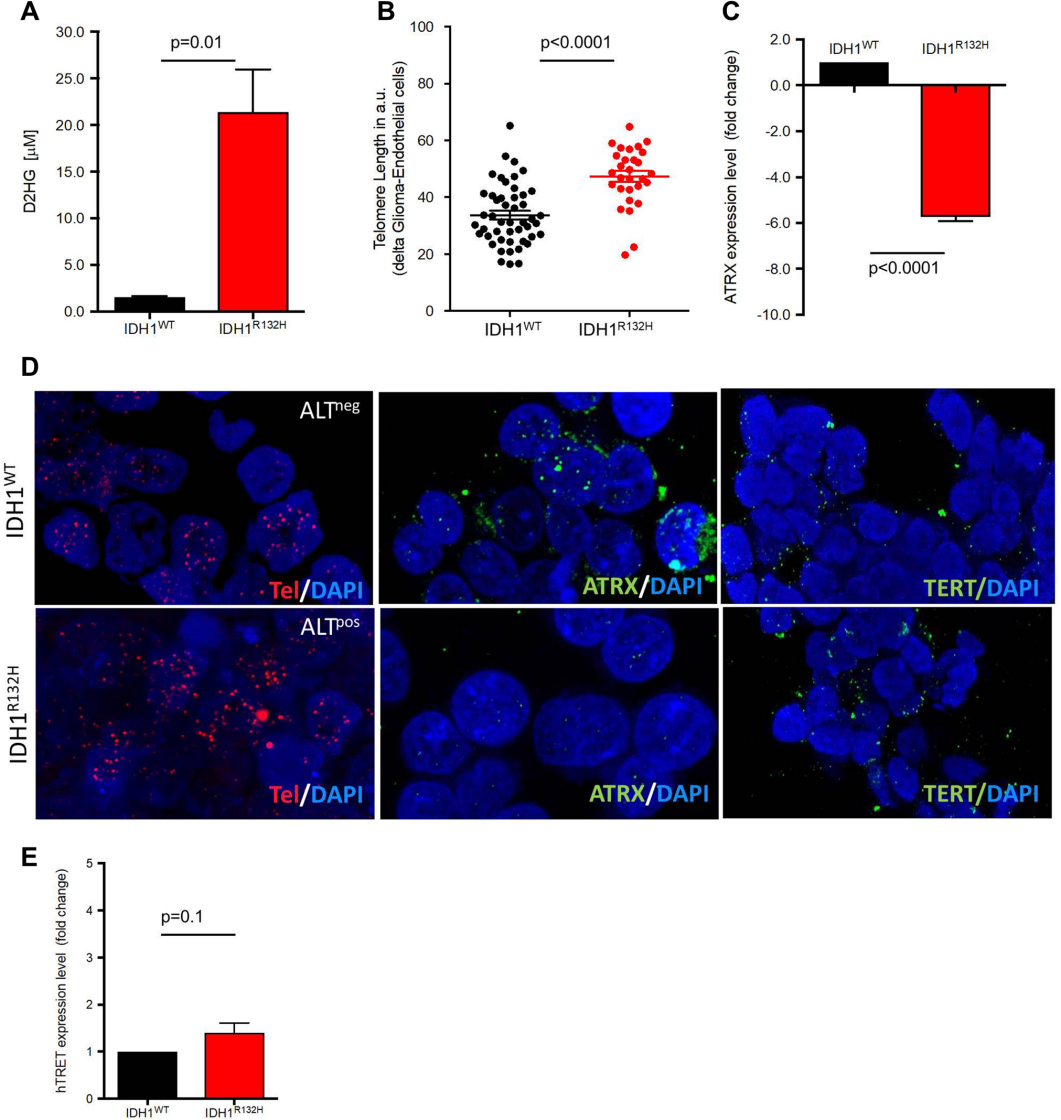
Telomerelength after induction of IDH1^R132H^ expression. **a** Production of the metabolite (inM) in the glioma cell line LN319 after the doxycycline-induced overexpression of IDH1^R132H^ and control conditions. D2HG was measured in the medium supernatant after three population doublings in culture. **b** Telomere length in the doxycycline induced IDH1^WT^ and IDH1^R132H^ cell lines, after nine population doublings in culture (IDH1^WT^ = 33.76 ± 1.54 a.u, n = 48 vs. IDH1^R132H^ = 47.3 ± 1.93 a.u, n = 29). **c** Fold change of ATRX mRNA expression after doxycycline treatment and nine population doublings as compared to untreated controls. **d** Representative images of Q-FISH stained doxycycline induced IDH1^WT^ (upper row) and IDH1^R132H^ cell lines (lower row). All images correspond to cell culture after 14 population doublings. The presence of ALT (left panel: telomere FISH (red), DAPI counterstaining), ATRX protein expression (middle panel: ATRX immunofluorescence (green), DAPI counterstaining), and TERT expression (right panel: TERT immunofluorescence (green), DAPI counterstaining) is given (magnification 756x). **e** TERT expression levels (fold change) at the mRNA level of doxycycline-induced IDH1^WT^ and IDH1^R132H^ cell lines after nine population doublings in culture

To understand the role of IDH1^R132H^ mutations in regulating CS-TL in glioma, we used a doxycycline-inducible GBM cell line (LN319) expressing IDH1^WT^ and IDH1^R132H^. The cell line was previously described and the presence of the IDH1^R132H^ mutation was confirmed by sequencing [39]. IDH^R132H^ expression induced by doxycycline significantly stimulated D2HG synthesis (p = 0.01; Fig. 4a), and compromised proliferation and clonogenicity (Supplementary Figure S3A-B). In this in vitro model, induced IDH1^R132H^ overexpression also resulted in a significant increase in TL after nine population doublings (Fig. 4b; Supplementary Figure S3C) and a significant downregulation of ATRX at transcriptional level (Fig. 4c). Telomere FISH detected a significant increase in ALT^pos^ cells in IDH1^R132H^ cells (Fig. 4d, left panels) and loss of ATRX protein expression (Fig. 4d) middle panels). Induced IDH^R132H^ overexpression neither altered the TERTp^mut^ status after short or long-term culture (Supplementary Figure S3D) nor significantly increased TERT mRNA expression level (Fig. 4e). Immunofluorescence for TERT protein expression with DAPI counterstaining (Fig. 4d, right panels) illustrated the TERT-independent increase of TL in IDH^R132H^ cells.

## Discussion

Maintenance mechanisms of telomeres are promising therapeutic targets and small molecule telomerase inhibitors are currently tested in clinical trials and filed for approval for the treatment of myeloproliferative syndromes [40, 41]. Exact understanding of telomere biology in glioma is therefore of high translational importance, given the completely diverse prognostic impact of TERTp mutations according to the IDH1 mutation status. Here we provide evidence suggesting that glioma subgroups with and without IDH1 mutations use different TMM and describe a new pathway linking the IDH1^R132H^ mutations to ALT.

One major limitation of published studies on telomere biology in solid cancer was missing availability of techniques that allow the determination of TL on a single cell level. In most studies performed so far [42–44], TL in tumor samples was actually derived from a mixture of vessels, immune cells and normal brain or tumor cells. This impaired significantly the validity and robustness of such studies. Furthermore, these studies rarely controlled for inter-individual (mostly genetic) variability of TL or the presence of ALT. In our study, we developed a modified immuno-Q-FISH technique for the determination of the TL in glioma cells at a single cell level, thus allowing to overcome some of these limitations not only by measuring tumor cells individually but also by using endogenous endothelial cells as non-malignant controls. Using this technique, we could show an increased CS-TL in IDH1^R132H^ mutated as compared to IDH1^WT^ tumors.

Concerning the role of ARTX mutations in gliomas [21, 23, 26–28], we confirmed the association with survival[45] and with ALT[21] as well as the association among ALT, ATRX expression loss, and IDH1^R132H^. However, the samples size of our cohort does not allow sound conclusions the prognostic relevance of the different TMM and the lacking association of ALT and TERTp mutations in the multivariate analysis have to be interpreted with caution. In line with Heidenreich et al. [42], our data supports the evidence that TERTp^mut^ are associated with shorter TL in gliomas.

Here we showed that the IDH1^R132H^ mutation is directly associated with a lack of ATRX expression and consequently, ALT as described previously [46]. In our sample, all tumors with ALT bear IDH1^R132H^ mutations and lost ATRX expression. Together with the inverse association of ALT with TERTp^mut^, our data suggests that ALT is the major TMM in IDH1^R132H^ astrocytoma. Conversely, TERTp mutations appear to be the crucial TMM in IDH^WT^ astrocytoma.

**Fig. 5:**
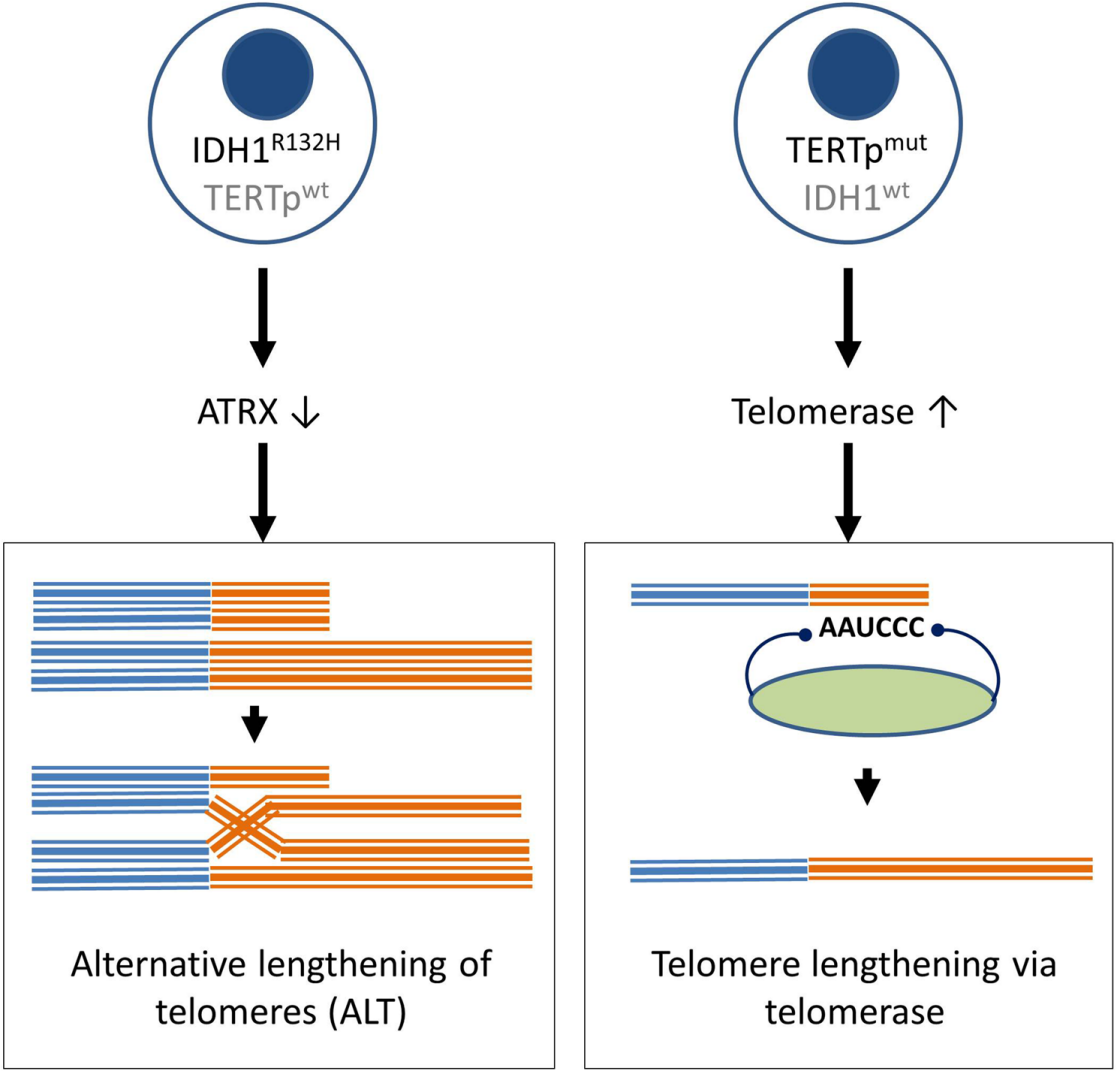
Overview of the two mechanisms of telomere maintenance. **a** Acquisition of IDH1^R132H^ mutation leads to reduced ATRX expression and ALT. **b** Acquisition of TERTp^mut^ mutation results in increased telomerase expression and telomere elongation via telomerase

Our data suggests a dichotomy of mechanisms in astrocytoma depending on the presence of IDH1 mutations (Fig. 5). In one tumor with TERTp mutations and ALT, the co-existance of two distinct TMM in IDH1^R132H^ cells of the same tumor (ALT and telomerase-dependent mechanisms) may exist, similar to the mosaic hypothesis previously suggested for other tumor types, e.g. sarcomas [47]. The proposed dichotomy indicates that treatment strategies targeting telomere maintenance, e.g. treatment with telomerase-inhibitors or ALT targeted treatment such e.g. PARP inhibitors or ATRX directed drugs [48, 49], must be personalized to the patient according to TMM. It is likely that the use of telomerase inhibitors can be ineffective or even detrimental in treating patients with IDH^R132H^ gliomas. Thus, the determination of ALT could be usefull as predictive marker to identify patients not responding to telomerase inhibitors.

We also showed that overexpression of IDH1^R132H^ in glioma cells in vitro result in a phenotype that fully mimicked all phenomena observed in the patient samples. Overexpression of IDH1^R132H^ in the glioma cells resulted in D2HG production, decreased proliferation in vitro, loss of ATRX expression in vitro and ALT. Reduced ATRX expression due to IDH1^R132H^ overexpression suggests that IDH1^R132H^ alone is sufficient to diminish ATRX expression and thereby induce ALT. However, the importance and functional relevance of this mechanism needs to be further confirmed in vivo. Further, it remains to be clarified, why IDH^R132H^ mutated glioma cells favor ATRX mutations, although there is a second, alternative pathway to suppress ATRX.

An obvious mechanism linking IDH1^R132H^ phenotype to the loss of ATRX in human glioma may be the existence of a typical hypermethylation/CpG island methylation of the ATRX gene. Our data partially supports the results found by Ohba et al. [50], who showed that IDH1 mutations do not select for or induce ATRX mutations or TERTp^mut^. However, we found no significant reactivation of TERT.

In conclusion, we found that ALT is the major TMM in IDH1^R132H^ astrocytomas and that IDH1^R132H^ mutations can directly suppress ATRX expression resulting in ALT.

## Electronic supplementary material

Below is the link to the electronic supplementary material.

Supplementary material 1 (JPEG 291 kb) **Supplementary Figure S1. (A)** Representative images of the immunofluorescence-based strategy used for CS-TL quantification in single tumor cells; tumor areas were identified by H&E staining, then sections were Q-FISH stained in combination with alfa-SMA immunofluorescence for identification of vessel cells (non-tumor cells that served as internal control). DAPI was used for nuclear counterstain of all cells (magnification 756x). **(B)** Telomere length in glioma patients according to WHO tumor grade. **(C)** Kaplan Meier survival curve of astrocytoma patients stratified based on IDH1 mutational status.

Supplementary material 2 (JPEG 166 kb) **Supplementary Figure S2. (A)** Representative chromatograms showing TERT promoter status (wildtype vs. C228T or C250T mutation) in the glioma tumors after Sanger sequencing.

Supplementary material 3 (JPEG 406 kb) **Supplementary Figure S3. (A)** Representative images of the colony-forming unit assay of non-induced (-) and doxycycline-induced (+) IDH1^WT^ and IDH1^R132H^ cell lines (scale: 1.7 cm). **(B)** Representative images of the agar assay of non-induced (-) and doxycycline induced (+) IDH1^WT^ and IDH1^R132H^ cell lines (magnification 50x). **(C)** Representative images of Q-FISH stained doxycycline induced IDH1^WT^ and IDH1^R132H^ cell lines after one (PD1) and five (PD5) population doublings in culture (magnification 756x); **(D)** Representative chromatograms showing absence of TERTp^mut^ by Sanger sequencing in doxycycline induced IDH1^WT^ and IDH1^R132H^ cell lines after three (PD3) and 27 (PD27) population doublings in culture.
