# Supplementary figures and images for: Alternative lengthening of telomeres is the major telomere maintenance mechanism in astrocytoma with isocitrate dehydrogenase 1 mutation
Source: J Neurooncol. 2020 Jan 20;147(1):1–14. doi: 10.1007/s11060-020-03394-y (PMC7076064; doi:10.1007/s11060-020-03394-y)

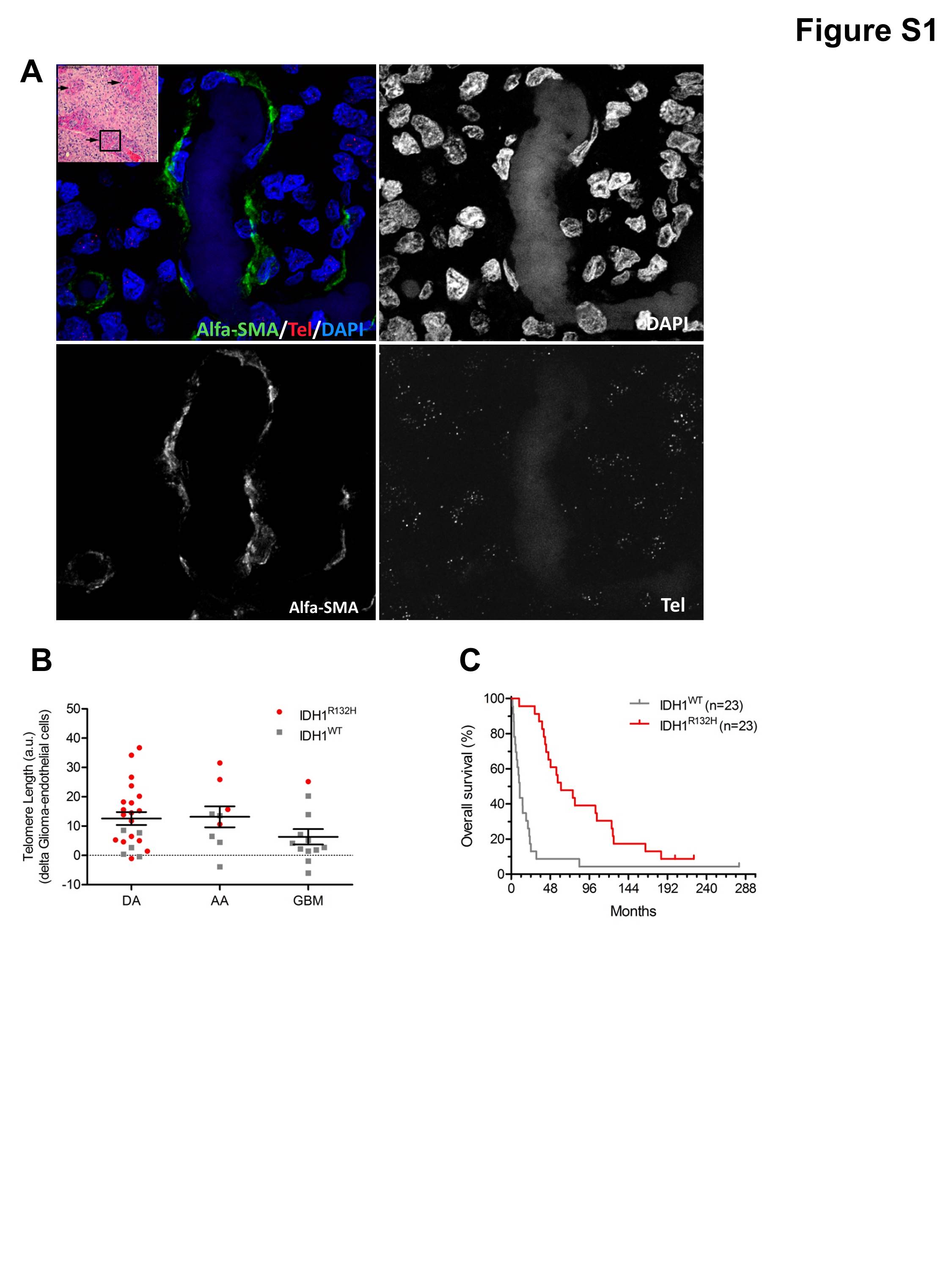

Supplement: Supplementary file 1 — Supplementary material 1 (JPEG 291 kb) Supplementary Figure S1. (A) Representative images of the immunofluorescence-based strategy used for CS-TL quantification in single tumor cells; tumor areas were identified by H&E staining, then sections were Q-FISH stained in combination with alfa-SMA immunofluorescence for identification of vessel cells (non-tumor cells that served as internal control). DAPI was used for nuclear counterstain of all cells (magnification 756x). (B) Telomere length in glioma patients according to WHO tumor grade. (C) Kaplan Meier survival curve of astrocytoma patients stratified based on IDH1 mutational status. [file 11060_2020_3394_MOESM1_ESM.jpg]

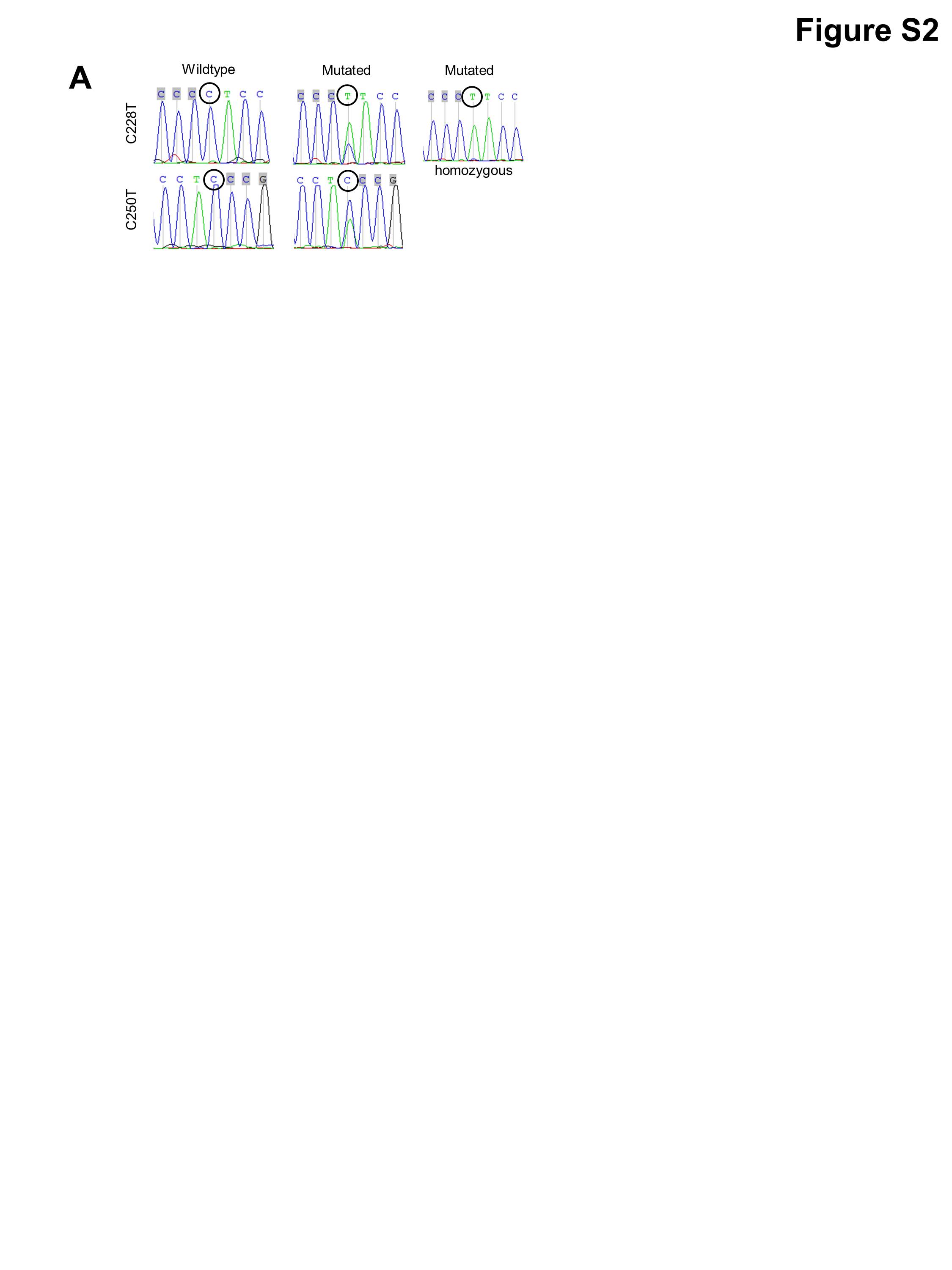

Supplement: Supplementary file 2 — Supplementary material 2 (JPEG 166 kb) Supplementary Figure S2. (A) Representative chromatograms showing TERT promoter status (wildtype vs. C228T or C250T mutation) in the glioma tumors after Sanger sequencing. [file 11060_2020_3394_MOESM2_ESM.jpg]

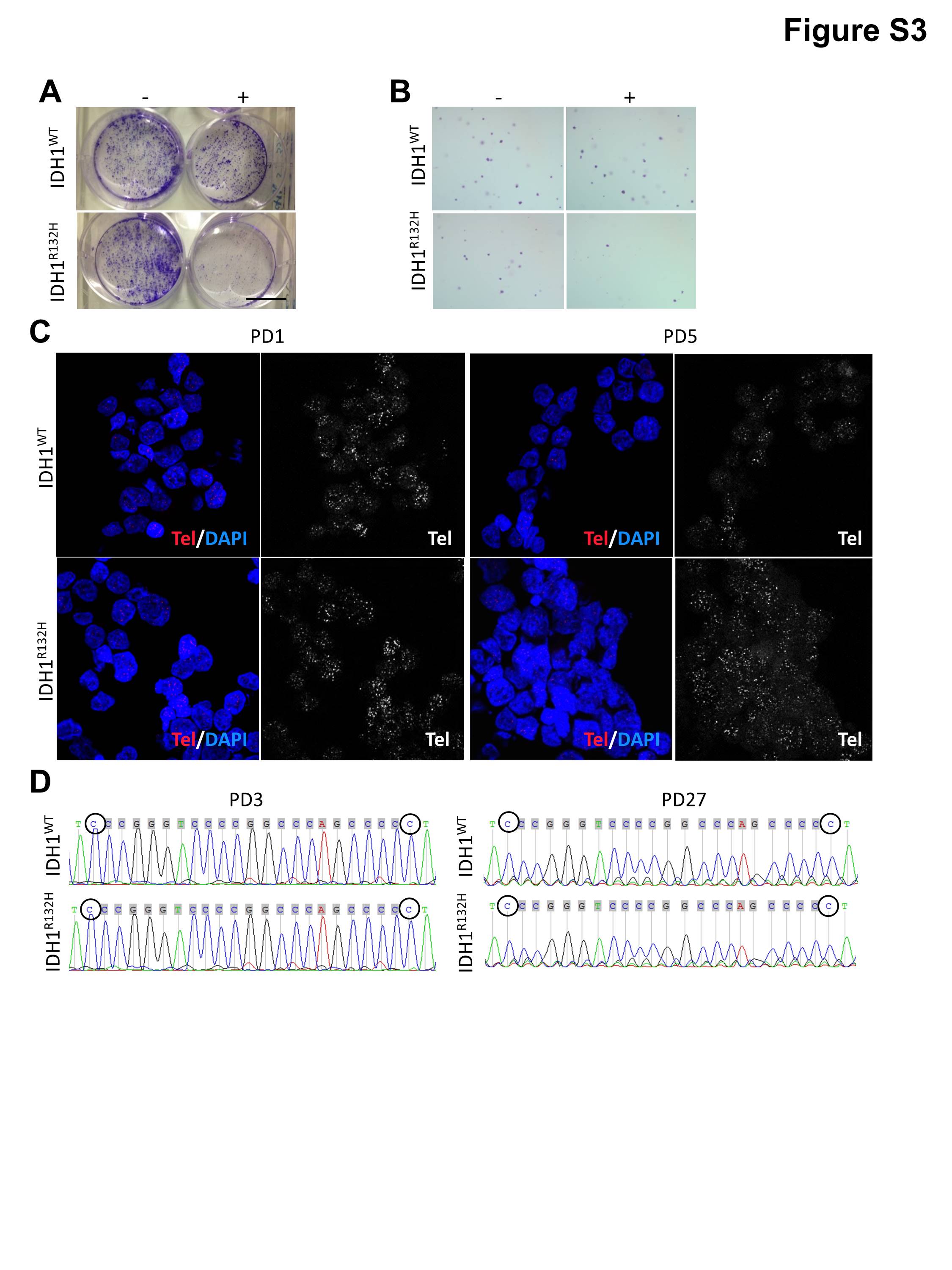

Supplement: Supplementary file 3 — Supplementary material 3 (JPEG 406 kb) Supplementary Figure S3. (A) Representative images of the colony-forming unit assay of non-induced (-) and doxycycline-induced (+) IDH1WT and IDH1R132H cell lines (scale: 1.7 cm). (B) Representative images of the agar assay of non-induced (-) and doxycycline induced (+) IDH1WT and IDH1R132H cell lines (magnification 50x). (C) Representative images of Q-FISH stained doxycycline induced IDH1WT and IDH1R132H cell lines after one (PD1) and five (PD5) population doublings in culture (magnification 756x); (D) Representative chromatograms showing absence of TERTpmut by Sanger sequencing in doxycycline induced IDH1WT and IDH1R132H cell lines after three (PD3) and 27 (PD27) population doublings in culture. [file 11060_2020_3394_MOESM3_ESM.jpg]
